# Ramalina capitata (Ach.) Nyl. acetone extract: HPLC analysis, genotoxicity, cholinesterase, antioxidant and antibacterial activity

**DOI:** 10.17179/excli2017-301

**Published:** 2017-05-11

**Authors:** Ivana Zrnzevic, Miroslava Stankovic, Vesna Stankov Jovanovic, Violeta Mitic, Aleksandra Dordevic, Ivana Zlatanovic, Gordana Stojanovic

**Affiliations:** 1 Department of Chemistry, Faculty of Science and Mathematics, University of Niš, Višegradska 33, 18000 Niš, Serbia; 2 Nuclear Facilities of Serbia, Mike Petrovica Alasa 12-14, 11351 Vinca, Serbia

**Keywords:** Ramalina capitata acetone extract, micronucleus test, antioxidant activity, cholinesterase inhibition, antimicrobial activity, chemical composition

## Abstract

In the present investigation, effects of *Ramalina capitata* acetone extract on micronucleus distribution on human lymphocytes, on cholinesterase activity and antioxidant activity (by the CUPRAC method) were examined, for the first time as well as its HPLC profile. Additionally, total phenolic compounds (TPC), antioxidant properties (estimated via DPPH, ABTS and TRP assays) and antibacterial activity were determined. The predominant phenolic compounds in this extract were evernic, everninic and obtusatic acids. Acetone extract of *R. capitata* at concentration of 2 μg mL^-1^ decreased a frequency of micronuclei (MN) for 14.8 %. The extract reduces the concentration of DPPH and ABTS radicals for 21.2 and 36.1 % (respectively). Values for total reducing power (TRP) and cupric reducing capacity (CUPRAC) were 0.4624 ± 0.1064 μg ascorbic acid equivalents (AAE) per mg of dry extract, and 6.1176 ± 0.2964 μg Trolox equivalents (TE) per mg of dry extract, respectively. The total phenol content was 670.6376 ± 66.554 μg galic acid equivalents (GAE) per mg of dry extract. Tested extract at concentration of 2 mg mL^-1^ exhibited inhibition effect (5.2 %) on pooled human serum cholinesterase. The antimicrobial assay showed that acetone extract had inhibition effect towards Gram-positive strains. The results of manifested antioxidant activity, reducing the number of micronuclei in human lymphocytes, and antibacterial activity recommends *R. capitata* extract for further *in vivo* studies.

## Introduction

Lichens are a slow growing complex of organisms composed of fungi and algae that are living in a mutually beneficial (symbiotic) relationship. The body of lichen is formed of fungus which contains one or more types of alga or cyanobacterium (occasionally both) (Temina et al., 2010[26]). As far as is known, every lichen species produces some unique secondary compounds - lichen substances, including depsides, depsidones, naphthoquinones, anthraquinones, pulvinates, chromones and dibenzofurans, often in remarkably large quantities (Manojlović et al., 2010[13]). These secondary metabolites are produced through polyketide, shikimic acid, and mevalonic acid pathways (Elix and Stocker-Wörgotter, 1996[6]).

The usage of many different lichens for many years in the traditional medicine, food, perfumery and cosmetics industries (Richardson, 1974[18]), was later justified by numerous researches that confirmed their great diversity of ecological and biological activities, including antioxidant, antibiotic, antimycobacterial, antiviral, antiinflammatory, analgesic, antipyretic, antiproliferative and cytotoxic effects (Dayan and Romagni, 2001[4]; Huneck, 1999[10]).

*Ramalina capitata* belongs to the family *Ramalinacea* that contains over 240 species. The genus *Ramalina* has a worldwide distribution, from coastal to alpine habitats, growing on different substrate: on bark, wood, soil (very rare) and rock, and belongs to the group of greenish fruticose lichens. Thallus of this lichen is in the form of flattened, strap-ike branches (Sharnoff, 2014[19]).

Usnic acid is the most common subject of research in *Ramalina* species, because of its diversity of biological activities (Cansaran et al., 2007[3]). In addition to this component that is located in the cortex of thallus, other compounds, including salazinic acid, divaricatic acid, sekikaic acid, homosekikaic acid, protocetraric acid, orsellinic acid, norstictic acid and lecanoric acid are present in the medulla of thallus (Aptroot and Bungartz, 2007[2]). Among *Ramalina* species *R. capitata* has been the subject of several studies. Cansaran et al. (2007[3]) examined antimicrobial activity, while Halici et al. (2011[9]) examined gastroprotective and antioxidative effects of *R. capitata* extracts. GC-MS profile of different *R. capitata* extract was published (Zrnzević et al., 2015[30], 2017[29]), as well as content of usnic acid (Cansaran et al., 2007[3]).

Taking into account a few information on the chemical composition and biological activity of *R. capitata*, the aim of the present work was to determine the secondary metabolites of *R. capitata* acetone extract by HPLC-DAD and to evaluate the effect on micronucleus distribution on human lymphocytes, effects on cholinesterase activity, total phenolic content, antioxidant activity (total reducing power, cupric reducing, DPPH and ABTS scavenging radical capacity) and antibacterial (against two Gram-positive and three Gram-negative bacteria) activities. To the best of our knowledge, HPLC analysis as well as effect on micronucleus distribution on human lymphocytes and effect on cholinesterase activity* of R. capitata* acetone extract has not been previously determined.

## Materials and Methods

### Lichen material

The lichen sample of *Ramalina capitata *(Ach.) Nyl. was collected in May 2015, at the mountain Stara planina - “Old Mountain” (mountain peak Babin zub - 1650 m above sea level; Serbia) from the population growing on rock habitat (red sandstone, silicates). The lichen *R. capitata* was authenticated by Dr. Bojan Zlatković. The voucher specimen of the lichen has been deposited in the Herbarium collection at the Department of Biology and Ecology, Faculty of Science and Mathemtaics, University of Niš under the acquisition number 9374. The lichen material (lichen thallus) was air dried without exposure to direct sunlight for 10 days and stored at room temperature (25 °C ± 2 °C), before extract preparation.

### Preparation of lichen extracts 

The extractions of the powdered lichen sample (10 g) were performed in triplicate (with 75 mL of acetone) using ultrasound bath (UZK 8; Maget, Bela Palanka, Serbia) for 30 min; after that, the extract was left in the dark (room temperature) for a period of five days. Dry residue of the extract was obtained using a rotary evaporator with the water bath set at 40 °C. The extract yield was 6.4 ± 0.5 %. 

### HPLC analysis

HPLC analysis was performed on an Agilent, Zorbax Eclipse XDB-C18, 5 μm, 4.6×150 mm column, by using a liquid chromatograph (Agilent 1200 series), equipped with a diode array detector (DAD), Chemstation Software (Agilent Technologies), a quaternary pump, an online vacuum degasser, an autosampler and a thermostatted column compartment. The mobile phase, methanol/water/formic acid = 80/20/0.2 (v/v/v), was pumped at a flow-rate of 0.5 mL min^-1^, the injection volume was 5 μL (concentration 10 mg of the dry extract per 1 mL of acetone, filtered through 0.45 µm filter), at 25 °C. The spectra were acquired in the range 190-400 nm and chromatograms plotted at 254 nm. Identification was conducted using retention time and UV spectra.

### Cytokinesis - block micronucleus assay (CBMN)

Cytokinesis - block micronucleus assay was performed as previously described (Fench and Morley, 1993[7]; Stojanović et al., 2013[25]). The cell culture lymphocytes were treated with 1.0, 2.0 and 3.0 μg mL^-1^ of the examined acetone extracts. Amifostine WR-2721 (98 % S-2 [3-aminopropylamino]-ethyl-phosphothioic acid; Marligen-Biosciences) at concentration of 1 μg mL^-1^ was used as a positive control. Three experiments were performed for each sample. The results are expressed as the means ± standard deviation (SD).

The statistical analysis was performed using Origin software package version 7.0. The statistical significance of difference between the data pairs was evaluated by analysis of variance (one-way ANOVA) followed by the Tukey test. Statistical difference was considered significant at p < 0.01 and *p *< 0.05.

### Total phenolic content and antioxidant activity 

Total phenolic content (TPC) and four antioxidant assays:DPPH and ABTS scavenging radical capacity, CUPRAC (cupric reducing antioxidant capacity) and TRP (total reducing power) were performed as previously described (Dimitrijević et al., 2015[5]; Re et al., 1996[17]). All spectrophotometric assays were conducted on a double beam UV/VIS spectrophotometer Perkin Elmer lambda 15 (Massachusetts, USA). For all above mentioned experiments concentration of sample solution was 15 mg of extract per mL of methanol. All analyses were performed in triplicate. Results are presented as mean ± standard deviation (SD).

### Cholinesterase activity

Assessment of extract effect on cholinesterase activity was performed as previously described (Stankov-Jovanović et al., 2015[22]). Activity was measured spectrophotometrically using a Konelab 20 analyzer (Thermofisher Scientific, Helsinki, Finland) with flow thermostatted cells, length 7 mm (at wavelength 405 nm). Sample concentration was 10 mg of dry extract per 1 mL of DMSO. Solution of neostigmine bromide at a concentration of 200 μg mL^-1^ was used as reference standard.

### Antibacterial activity

Antibacterial activity was evaluated against two Gram-positive* (Bacillus spizizenii *ATCC 6633 and *Staphylococcus aureus *ATCC 6538) and three Gram-negative bacteria (*Escherichia coli *ATCC 8739, *Pseudomonas aeruginosa *ATCC 9027 and *Salmonella abony *ATCC 6017). Analysis was performed according to the NCCLS (1997[15]). Each test was performed in triplicate. Sample concentration was 1 mg per disk (diameter 12 mm). Streptomycin and chloramphenicol were used as a positive control, at concentration of 10 µg and 30 µg per disk, respectively. 

## Results and Discussion

### HPLC-DAD analysis

Results of HPLC-DAD analysis are given in Table 1(Tab. 1).

**Figure 1:**
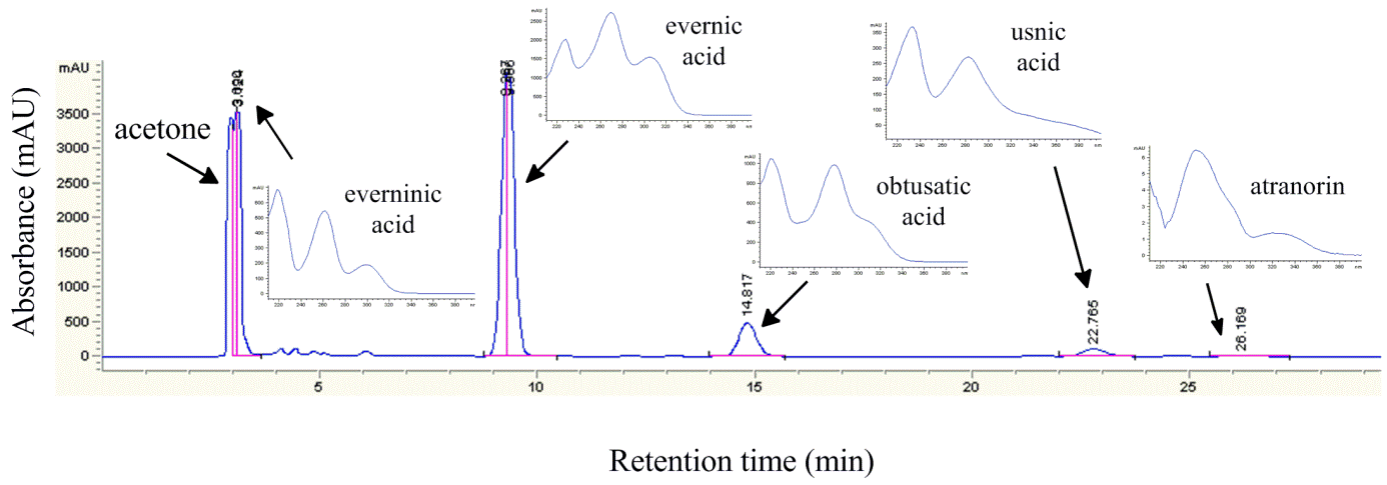
HPLC Chromatogram of *R. capitata *acetone extract and UV spectra of identified constituents

The HPLC chromatogram as well as UV spectra of identified components of *R. capitata* acetone extract are given on Figure 1(Fig. 1). 

**Figure 2:**
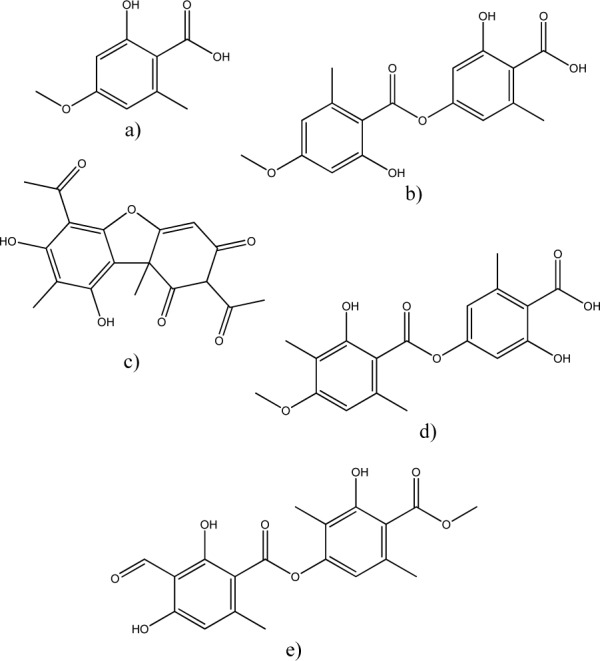
The chemical structures of *R. capitata *acetone extract constituents: a) everninic acid; b) evernic acid; c) usnic acid; d) obtusatic acid; e) atranorin

Five metabolites: everninic acid, evernic acid, obtusatic acid, usnic acid and atranorin, were identified in lichen *R. capitata*. Evernic acid exhibited the most intense peaks in the *R. capitata* HPLC chromatogram. Compounds identified in the extract belong to depsides (evernic acid, obtusatic acid and atranorin) and cleavage product of depsides (everninic acid), while usnic acid is lichen metabolite with dibenzofuran structure (Figure 2(Fig. 2))*.*

Similar to our results, the previous studies (Moreira et al., 2015[14]) showed that usnic acid, evernic acid, obtusatic acid and atranorin were commonly found in many species of genus *Ramalina*. According to Cansaran et al. (2007[3]) the acetone extract of *R. capitata* contained around 1.3 % of usnic acid, while *Ramalina fastigiata* yielded the highest usnic acid content with the value of 3.23 %. The most represented compounds in the ether-soluble fraction of *R. capitata* methanol extract were orcinol (22.9 %) and its monomethyl ether (30.9 %) (Zrnzević et al., 2015[30]), while the main identified components in the ether, ethyl acetate and dichloromethane extracts of *R. capitata* were everninic acid (24.7, 33.7 and 22.2 %), orcinol (25.8, 16.7 and 11.9 %), orcinol monomethyl ether (11.6, 7.6 and 4.8 %), 3-methylorsellinic acid (10.2, 7.1 and 9.0 %) and usnic acid (4.4, 8.2 and 25.8 %), respectively (Zrnzević et al., 2017[29]). Based on fact that everninic acid was the second most abundant component in present examined extract (27.2 %), it could indicate that it is not only product of the degradation at high temperature during GC analysis, but also an integral part of the extract.

### Cytokinesis - block micronucleus assay (CBMN)

In this study, *in vitro* protective effect of the extract (at concentrations of 1.0, 2.0 and 3.0 μg mL^-1^) on micronuclei formation in peripheral human lymphocyte cultures was examined using the cytochalasin-B blocked micronucleus (MN) assay. The frequencies and distribution of MN in human lymphocytes were scored, and the results are presented in Table 2(Tab. 2).

The cell cultures treated with amifostine (radioprotectant, previously known as WR- 2721) at concentration of 1.0 μg mL^-1^ gave a decrease in the MN frequency of 11.4 % comparing to control cell cultures (statistically significant, p<0.05). Among the tested extract of *R. capitata* at concentration of 1.0, 2.0, and 3.0 μg mL^-1^, the highest activity was at concentration of 2.0 μg mL^-1^ that gave a decrease in the MN frequency of 14.8 % which was a higher effect than amifostine. The extracts at concentration of 1.0 μg mL^-1^ and 3.0 μg mL^-1^ caused slightly decrease of the MN frequency (6.7 % and 4.2 %, respectively), that is less effective than amifostine. CBPI (cytokinesis-block proliferation index) was used to determine the effect of tested extracts on cell proliferation. Comparison of the CPBI values of untreated cells, amifostine and extract, we found that there was an inhibitory effect on lymphocyte proliferation of tested extract of *R. capitata*. Approximately equal rate of cells proliferation is important for the validity of the results of micronucleus test.

MN assay provides a measure of both chromosome breakage and chromosome loss, and damaged DNA can lead to aneuploidy and/or chromosomal instability, which is believed to be a major contributor to cancer progression (Koparal et al., 2006[11]). Since the number of micronuclei serves as an indicator of DNA damage, these results indicate that examined extract of *R. capitata* at concentration of 2.0 μg mL^-1^ protects DNA. Previously, Koparal et al. (2006[11]) investigated cytotoxic and genotoxic activities of the lichens *Ramalina farinacea *and* Cladonia foliacea* and suggested that usnic acid (a main component of lichens) was non-genotoxic shown by the absence of MN induction in human lymphocytes. Similary, the genotoxic effects of methanol, acetone, n-hexane and ether extracts of *Pseudovernia furfuracea* lichen were ascertained by MN tests in human whole blood cultures. According to results of this study, it was established that these lichen extracts had also no genotoxic effect (Türkez et al., 2010[27]). 

### The total phenolic content and antioxidant activity

The antioxidant potential of acetone extract of *R. capitata* was evaluated by determining its total phenolic content (TPC), its ability for a DPPH and ABTS radical scavenging, total reducing power (TRP) and cupric reducing antioxidant capacity (CUPRAC). 

The total amount of phenolic compounds was determined as gallic acid equivalent (GAE) using an equation obtained from a standard gallic acid graph. The value of the total phenolics contents (TPC) of the *R. capitata* acetone extract was high and amounted 670.6376 ± 66.554 µg GAE mg^-1^. The obtained value for the total phenol content are considerably higher than those previously published for acetone extract of *Ramalina dumeticola* (101.62 ± 3.51 mg GAE per g dry extract) (Gunasekaran et al., 2016[8]). Likewise, lichen from the genus *Ramalina*, was investigated for its phenolic content. They also reported lower total phenolic content in *R. peruviana* (27.1 mg GAE per g dry extract) (Stanly et al., 2011[23]). Extract of the studied lichen showed a moderate DPPH and ABTS radical scavenging activity (21.25 % and 36.08 %, respectively). Previously, hexane extracts of *Ramalina roesleri* were tested for DPPH radical scavenging activity by Sisodia et al. (2013[21]). The DPPH radical scavenging activity ranged from 29.42 % to 87.90 %. Gunasekaran et al. (2016[8]) found that the value of DPPH scavenging activity for *Ramalina dumeticola* extract was 27.21 %, which is relatively similar to our results.

The value of the total reducing power ability (ability of antioxidants to reduce Fe(III) hexacyanate to Fe(II) hexacyanate which leads to an increase in the absorbance of the reaction mixtures; TRP) for *R. capitata* extract was 0.4624 ± 0.1064 µg ascorbic acid equivalents (AAE) per mg dry extract weight. Stojanović et al. (2010[24]) reported values of TRP from 21.63 to 96.9 μg of AAE per g of methanol extracts of some lichens (*Hypogymnia physodes, Evernia prunastri, Flavoparmelia caperata, Parmelia sulcata*). 

The CUPRAC method has several advantages over other antioxidant assays, primarily because is performed at physiological pH and its applicability to hydrophilic and lipophilic antioxidants (Apak et al., 2007[1]). The result obtained by CUPRAC method for *R. capitata *acetone extract was 6.1176 ± 0.2964 µg Trolox equivalents (TE) per mg dry extract. Recently, Zlatanović et al. (2017[28]) studied the cupric reducing antioxidant capacity of acetone extract obtained from the lichen, *Umbilicaria crustulosa*, and the obtained result was 19.7641 ± 0.0166 mg TE per mg dry extract. This value is higher than the result obtained from our sample. 

### Cholinesterase activity

The inhibition of *R. capitata* acetone extract on cholinesterase activity was in a dose-dependent manner. Extract at concentration of 1.0 mg mL^-1^ has manifested insignificant activation effect on cholinesterase to extent of 2.8 %, while more concentrated extract (10 mg mL^-1^) exhibited the slight inhibition effect (5.2 %) on pooled human serum cholinesterase. According to these results, it can be assumed that the increase of extracts' concentrations increases the ability to inhibit cholinesterase activity. In conducted experiment, neostigmin bromid (as standard cholinesterase inhibitor) inhibited cholinesterase to extent of 96.6 %. In previously research, Luo et al. (2013[12]) found that the extract of *Cladonia macilenta* showed a high cholinesterase inhibitory activity with 60.5 %, while Zlatanović et al. (2017[28]) found activity similar to ours for *Umbilicaria crustulosa* acetone extract. 

### Antibacterial activity 

The results of the antimicrobial potential of *R. capitata *acetone extract tested against five different bacteria are shown in Table 3(Tab. 3). The maximal inhibition zone for the tested microorganisms was 28 mm for *Bacillus spizizenii*. Slightly weaker antibacterial activity of tested extract was toward *Staphylococcus aureus*, with inhibition zone of 19 mm. The microorganisms *Escherichia coli*, *Pseudomonas aeruginosa *and *Salmonella abony *were resistant. In particular, the analysis showed that acetone extract had great inhibition effect towards Gram-positive bacteria and there is no effect on the Gram-negative bacteria. The antimicrobial activity was compared with the standard antibiotics, streptomycin and chloramphenicol. As shown in Table 3(Tab. 3), standards have stronger activity than tested samples.

The obtained results are not entirely in accordance with previously published. Namely, Cansaran et al. (2007[3]) have reported no activity toward *P. aeruginosa* and *S. aureus*, but moderate antimicrobial activity of acetone extracts of *R. capitata* to *E. coli* and *B. subtilis*. In the study conducted by Paudel et al. (2008[16]), the antibacterial potential of methanol extracts of *Ramalina terebrata, *it was shown that considerable antimicrobial activity was obtained against *B. subtilis *and *S. aureus*, but no activity was observed against *P. aeruginosa *and *E.coli.*

Based on these results, *R. capitata* acetone extract is similar to *Ramalina farinacea* (L.) Ach. by its composition (Shukla et al., 2010[20]). The manifested antioxidant activity, reducing the number of micronuclei in human lymphocytes, and the activity against *B. subtilis *and *S. aureus* bacteria recommends *R. capitata* extract for further *in vivo* studies.

## Acknowledgements

The authors acknowledge the Ministry of Education, Science and Technological Development of Serbia for financial support (Grant No 172047).

## Conflict of interest

The authors declare no conflict of interest.

## Figures and Tables

**Table 1:**
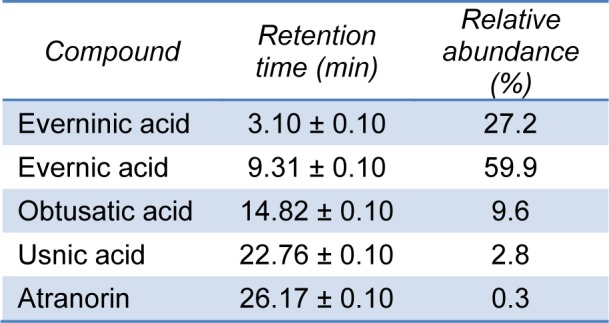
Retention time of the components of the *R. capitata* acetone extract and their relative abundance (% of the total HPLC peak area)

**Table 2:**
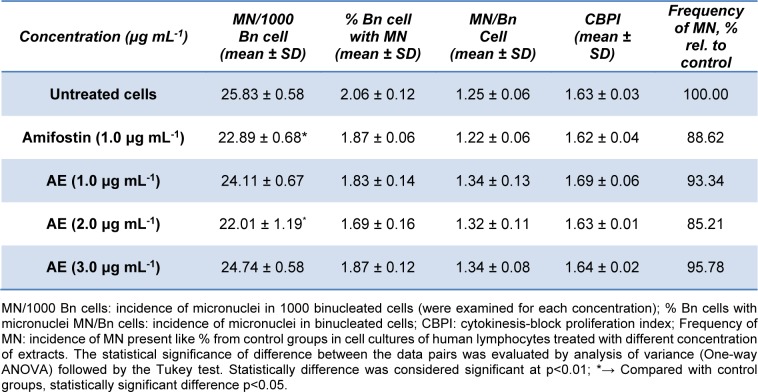
Incidence of MN, cytokinesis-block proliferation index, distribution of MN per cells and frequency of MN in cell cultures of human lymphocytes treated with different concentration of *R. capitata *acetone extract (AE)

**Table 3:**
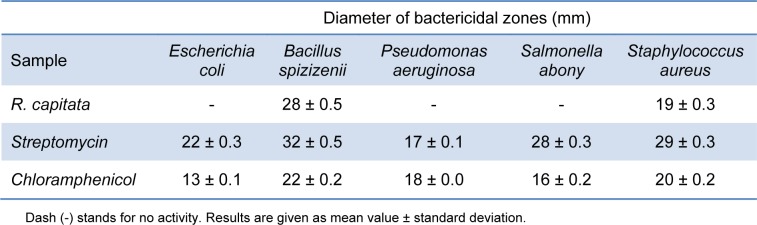
Bactericidal activity (in mm including disc diameter of 12 mm) of lichen extract (1 mg per disc) and antiobiotics, streptomycin (10 µg per disc) and chloramphenicol (30 µg per disc) toward five bacteria
